# Can we use baseline characteristics to assess which men with moderately symptomatic benign prostatic hyperplasia at risk of progression will benefit from treatment? A post hoc analysis of data from the 2-year CONDUCT study

**DOI:** 10.1007/s00345-016-1884-5

**Published:** 2016-06-22

**Authors:** Claus G. Roehrborn, Igor Oyarzabal Perez, Erik P. M. Roos, Nicolae Calomfirescu, Betsy Brotherton, Juan Manuel Palacios, Averyan Vasylyev, Michael J. Manyak

**Affiliations:** 1 Department of Urology, UT Southwestern Medical Center, 5323 Harry Hines Blvd, J8 142, Dallas, TX 75390-9110 USA; 2 Department of Urology, Mendaro Hospital, Mendaro, Gipuzkoa Spain; 3 Department of Urology, Antonius Hospital Sneek, Sneek, The Netherlands; 4 Urology Clinic, Uroandromed, Bucharest, Romania; 5 GlaxoSmithKline, Research Triangle Park, NC USA; 6 GlaxoSmithKline, Urology, Classic and Established Products, GlaxoSmithKline, Madrid, Spain; 7 GlaxoSmithKline Pharmaceuticals, GlaxoSmithKline, Kiev, Ukraine; 8 PAREXEL International, Durham, NC USA

**Keywords:** Benign prostatic hyperplasia, BPH Impact Index, Dutasteride, Lower urinary tract symptoms, Tamsulosin, Watchful waiting

## Abstract

**Purpose:**

To investigate (in a post hoc analysis of the 2-year CONDUCT study) the characteristics and clinical outcomes of men with moderately symptomatic benign prostatic hyperplasia (BPH) at risk of progression who benefitted from lifestyle changes alone.

**Methods:**

Patients were given lifestyle advice and randomized to a fixed-dose combination (FDC) of dutasteride and tamsulosin or watchful waiting (WW) and followed for 24 months. Patients in the WW group were escalated to tamsulosin if any follow-up International Prostate Symptom Score (IPSS) was equal or greater than the baseline value. Improvements in symptoms (change in IPSS) and quality of life [measured by BPH Impact Index (BII) and question 8 of the IPSS (IPSS-Q8)] were analysed in the FDC group, men who initiated tamsulosin (WW-TAM) and men who received no medical intervention (WW-no treatment) and the impact of baseline variables on IPSS determined.

**Results:**

The adjusted mean decrease in IPSS, BII and IPSS-Q8 at each post-baseline visit over 24 months appeared greater in the FDC (*n* = 369) and WW-no treatment groups (*n* = 144) than in the WW-TAM group (*n* = 229). IPSS improvements appeared similar in the FDC group and WW-no treatment subgroup, except in patients with the greatest degree of bother at baseline (BII 7–13).

**Conclusion:**

BII at baseline may be a more relevant indicator than symptom severity as to whether a patient with moderate symptoms should receive medical therapy or not.

## Introduction

More than 50 % of men aged ≥60 years have histological evidence of benign prostatic hyperplasia (BPH). BPH can be associated with benign prostatic enlargement (BPE), which can result in lower urinary tract symptoms (LUTS) such as weak stream and hesitancy; some men may also experience LUTS associated with storage, such as increased urgency, frequency and nocturia. These symptoms can significantly diminish a person’s quality of life (QoL) [1, 2].

The optimal time point to initiate medical therapy of BPH is a matter of debate [3]. Although guidelines recommend a conservative approach for men with minimal symptoms [4, 5], several observations argue that early intervention may be an appropriate option for some patients [6, 7].

The CONDUCT study was performed to investigate whether there was a difference in symptomatic improvement from baseline, as measured by the International Prostate Symptom Score (IPSS), among treatment-naïve men with moderately symptomatic BPH at risk of progression, who were given lifestyle advice and either immediate treatment with a fixed-dose combination (FDC) of dutasteride and tamsulosin or managed with watchful waiting (WW) with protocol-defined initiation of tamsulosin monotherapy if symptoms did not improve. The results of this study have previously been reported [8]. Notably, over one-third of men managed with WW in CONDUCT had symptomatic improvement without any pharmacological intervention, i.e. with lifestyle advice alone [8].

The mechanisms leading to an improvement in symptoms without pharmacologic intervention are poorly understood; however, condition-, patient- and lifestyle-specific factors could be involved [3]. To investigate this further, we report a post hoc analysis of CONDUCT 2-year data revealing the demographics, disposition and clinical outcomes of men who received immediate intervention with FDC, initiated tamsulosin monotherapy or received no pharmacological intervention and benefitted from lifestyle advice alone.

## Patients and methods

### Study design

The design of the CONDUCT study (GlaxoSmithKline FDC114615; NCT01294592) has been reported [8]. Eligible men [aged ≥50 years, confirmed clinical diagnosis of BPH, moderate LUTS (IPSS of 8–19), prostate volume ≥30 mL and total serum prostate-specific antigen (PSA) level of ≥1.5 ng/mL] were randomized 1:1 to self-administer a FDC of dutasteride 0.5 mg and tamsulosin 0.4 mg QD (Duodart^®^, GlaxoSmithKline), or WW with initiation of tamsulosin 0.4 mg QD if any IPSS after randomization was the same or greater than the baseline value. Lifestyle advice was provided at baseline to all patients.

Patients visited the clinic 4 weeks after randomization and at 13-week intervals thereafter, for up to 24 months. Patient-reported symptoms, symptom impact and BPH-related QoL were assessed at every visit using the last observation carried forward method. Data on adverse events were collected from first administration of study treatment (FDC or tamsulosin) until discontinuation and have been presented previously [8]. Safety data were not scheduled to be collected for patients in the WW group who received no drug.

### Post hoc data analyses

Symptomatic improvement, as measured by IPSS, was investigated in the FDC group, the WW subgroup who initiated tamsulosin (WW-TAM) and the WW subgroup who did not receive medical intervention (WW-no treatment). As the CONDUCT study was not powered to assess treatment effects in or across the WW-TAM and WW-no treatment subgroups, no formal statistical analyses were performed. Data from patients in the WW-TAM and WW-no treatment subgroups were supplemental and are provided for descriptive purposes only.

Data were summarised overall and by baseline subgroups as tabular displays and figures in terms of mean change from baseline in IPSS, BPH Impact Index (BII) and question 8 of the IPSS (IPSS-Q8). Changes in IPSS were also summarised according to the categorical subgroups of age (<65, ≥65 years) and BII (0–3, 4–6 or 7–13 at baseline).

## Results

### Patient demographics and disposition

In all, 742 patients were randomized into CONDUCT (369 and 373 in the FDC and WW groups, respectively). Of the 373 patients randomized to WW, 229 (61.4 %) initiated tamsulosin, mostly within the first 6 months of the study. Of 190 patients who started tamsulosin within 6 months of randomization, 125 (66 %) started in month 1, 45 (24 %) in month 3 and 20 (11 %) in month 6. Baseline characteristics were generally similar between the three groups (Table 1). Although the mean IPSS at baseline was the same for men in the WW-TAM and WW-no treatment subgroups, men in the WW-no treatment subgroup tended to be younger and less bothered by their symptoms at baseline.

**Table 1:** Baseline characteristics

Variable	FDC (*n* = 369)	WW-TAM (*n* = 229)	WW-no treatment (*n* = 144)	All (*N* = 742)
Age (years)	66.3	66.7	65.3	66.2
PSA (ng/mL)	3.9	3.8	3.6	3.8
Prostate volume (mL)	51.0	52.6	52.6	51.8
Mean IPSS	13.2	12.9	12.9	13.1
Mean BII score	4.8	4.7	4.0	4.6
BII 0–3 [*n* (%)]	123 (33)	75 (33)	68 (47)	266 (36)
BII 4–6 [*n* (%)]	132 (36)	94 (41)	37 (26)	263 (35)
BII 7–13 [*n* (%)]	114 (31)	60 (26)	39 (27)	213 (29)
IPSS-Q8	3.2	3.1	2.9	3.1

### Changes in IPSS, BII and IPSS-Q8

**Fig. 1:**
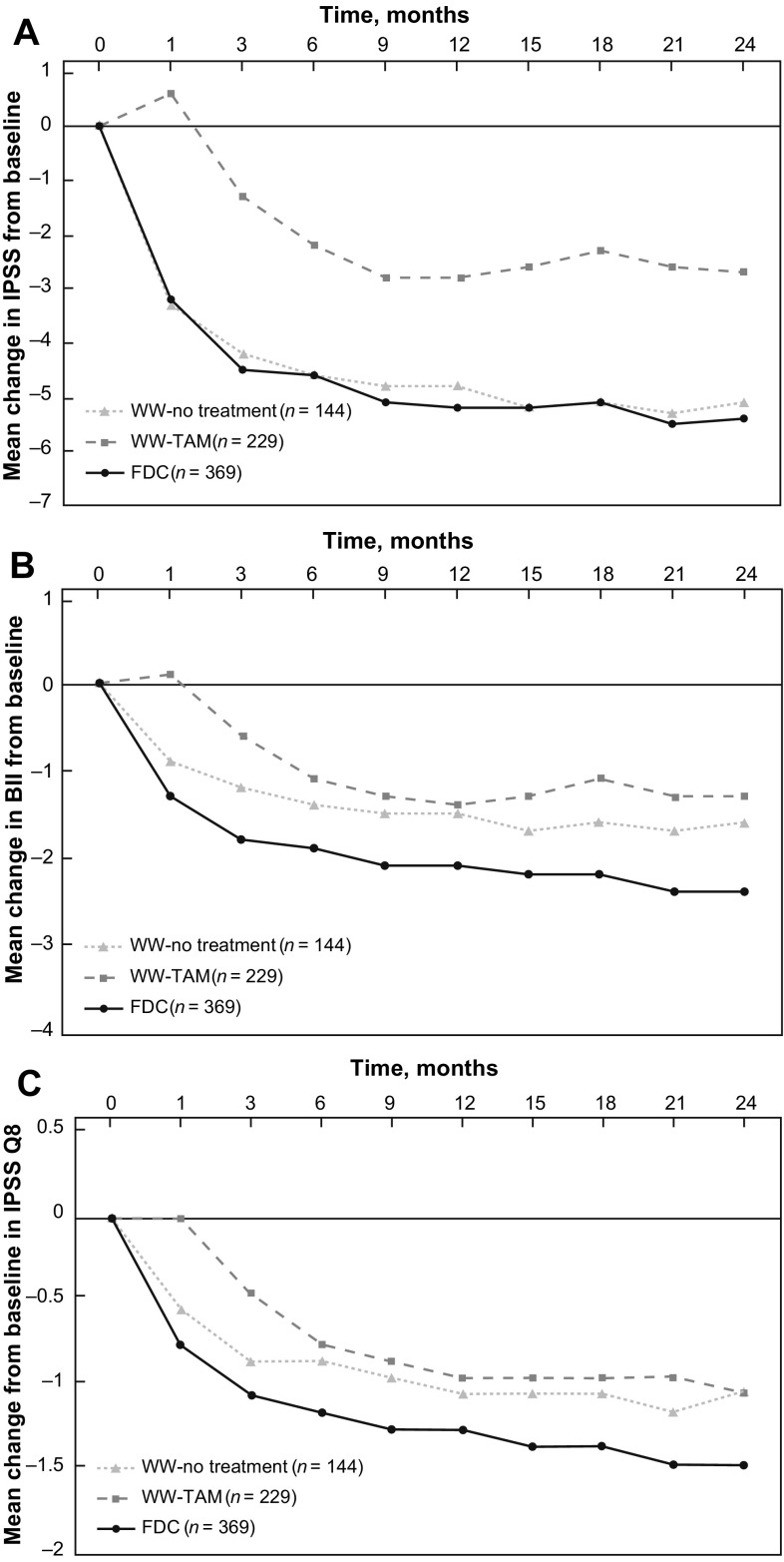
Mean change from baseline in **a** IPSS, **b** BII and **c** IPSS-Q8

The adjusted mean decrease (improvement) in IPSS at each post-baseline visit over 24 months appeared greater in the WW-no treatment subgroup than in the WW-TAM subgroup and very similar between the FDC group and WW-no treatment subgroup (Fig. 1a). At month 24, the mean change in IPSS was −5.6 for patients in the FDC group, −5.1 for patients in the WW-no treatment subgroup and −2.7 among patients who received tamsulosin.

The improvement in BII and IPSS-Q8 at each post-baseline visit over 24 months appeared greater in the FDC group than in either the WW-no treatment or the WW-TAM subgroups and greater in the WW-no treatment subgroup than in the WW-TAM subgroup (Fig. 1b, c).

### Impact of baseline variables on changes in IPSS

**Fig. 2:**
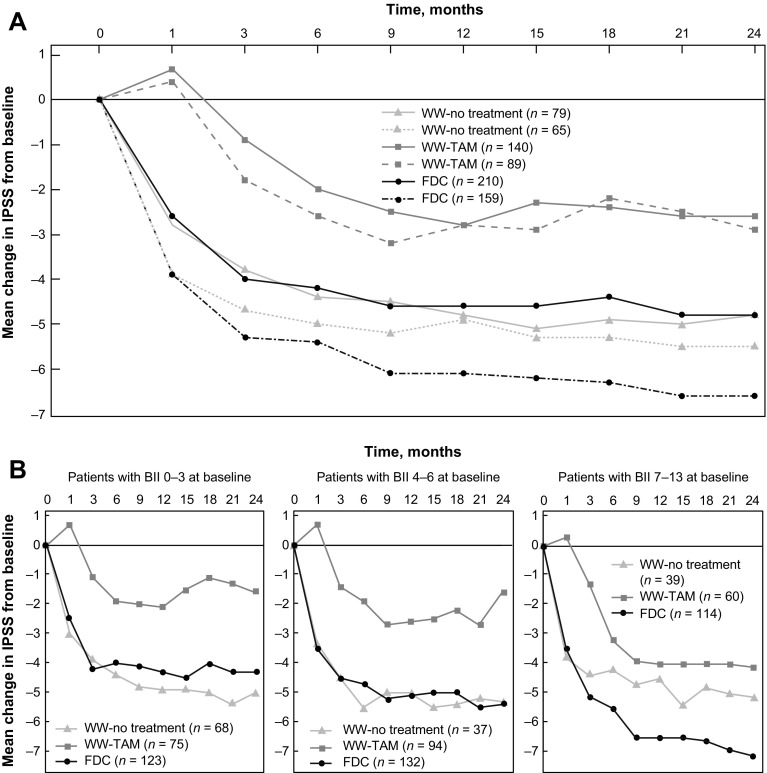
Mean change from baseline in IPSS according to **a** age (*solid line* ≥65 years; *broken line* <65 years) and **b** BII at baseline

The baseline characteristics of men aged ≥65 or <65 years of age were generally comparable (Table 2). Although men aged <65 years were more bothered by their urinary problems at baseline than older men (BII score of 5.1 vs 4.3), this did not translate into a difference in baseline IPSS. In the FDC and WW-no treatment subgroups, the mean IPSS was lower at each post-baseline visit in patients aged <65 years than in patients aged ≥65 years. Between baseline and month 24, the IPSS for patients aged <65 and ≥65 years improved by 6.6 and 4.8 points for patients in the FDC group, 5.5 and 4.8 points for patients in the WW-no treatment subgroup and 2.9 and 2.6 points in the WW-TAM subgroup (Fig. 2a; Table 3).

**Table 2:** Baseline characteristics of patients according to age

	≥65 years				<65 years			
	FDC	WW-no treatment	WW-TAM	All	FDC	WW-no treatment	WW-TAM	All
Patients (*n*)	210	79	140	429	159	65	89	313
Age (years)	71.8	70.6	71.5	71.5	59.0	58.9	59.3	59.1
Weight (kg)	79.78	81.63	82.5	81.01	83.37	84.02	85.26	84.05
Body mass index	26.93	27.87	27.87	27.39	27.73	28.07	28.13	27.91
Time since first LUTS (years)	4.2	4.0	4.5	4.3	3.4	3.0	2.7	3.1
Time since BPH diagnosis (years)	3.0	2.6	3.0	2.9	2.3	2.3	2.0	2.2
PSA (ng/mL)	3.9	3.7	4.0	3.9	3.8	3.5	3.5	3.6
IPSS	13.0	12.8	13.0	13.0	13.4	13.0	12.8	13.1
Prostate volume (mL)	51.6	56.9	55.2	53.7	50.3	47.5	48.6	49.3
BII	4.6	3.4	4.2	4.3	5.0	4.7	5.3	5.1
IPSS-Q8	3.1	2.7	3.0	3.0	3.2	3.1	3.2	3.2

**Table 3:** Mean IPSS change from baseline at 24 months according to baseline characteristics

Baseline characteristic	Mean change in IPSS from baseline at month 24 (patients, *n*)							
	Patients aged ≥65 years at baseline				Patients aged <65 years at baseline			
	FDC	WW-All	WW-no treatment	WW-TAM	FDC	WW-All	WW-no treatment	WW-TAM
≥ or <65 years	−4.8 (203)	−3.3 (216)	−4.8 (76)	−2.6 (140)	−6.6 (156)	−4.0 (152)	−5.5 (63)	−2.9 (89)
IPSS <13	−3.3 (92)	−1.7 (108)	−3.9 (42)	−0.4 (66)	−4.7 (77)	−3.4 (69)	−5.1 (31)	−2.0 (38)
IPSS ≥13	−5.9 (111)	−4.9 (108)	−5.9 (34)	−4.5 (74)	−8.5 (79)	−4.5 (83)	−5.9 (32)	−3.7 (51)
BII 0–3	−4.1 (69)	−2.7 (98)	−4.4 (43)	−1.4 (55)	−4.6 (49)	−4.2 (43)	−6.1 (23)	−2.0 (20)
BII 4–6	−4.5 (78)	−3.7 (77)	−5.9 (20)	−3.0 (57)	−6.9 (52)	−2.9 (53)	−4.5 (16)	−2.2 (37)
BII 7–13	−5.9 (56)	−4.0 (41)	−4.3 (13)	−3.9 (28)	−8.3 (55)	−4.9 (56)	−5.6 (24)	−4.3 (32)
IPSS Q8 <4	−4.4 (133)	−3.0 (146)	−4.7 (54)	−1.9 (92)	−5.4 (96)	−4.0 (96)	−5.4 (40)	−2.9 (56)
IPSS Q8 ≥4	−5.4 (70)	−4.1 (70)	−4.9 (22)	−3.7 (48)	−8.7 (60)	−4.1 (56)	−5.7 (23)	−3.0 (33)
Prostate volume <40 mL	−4.7 (67)	−3.6 (49)	−4.7 (17)	−3.0 (32)	−5.9 (54)	−4.2 (47)	−5.3 (21)	−3.3 (26)
Prostate volume ≥40 mL	−4.8 (135)	−3.3 (167)	−4.8 (59)	−2.4 (108)	−7.0 (102)	−3.9 (105)	−5.6 (42)	−2.8 (63)
PSA <3 ng/mL	−3.8 (78)	−4.2 (82)	−5.1 (31)	−3.6 (51)	−6.2 (69)	−4.3 (77)	−6.0 (31)	−3.1 (46)
PSA ≥3 ng/mL	−5.3 (125)	−2.8 (134)	−4.6 (45)	−1.9 (89)	−7.0 (87)	−3.7 (75)	−5.0 (32)	−2.8 (43)

Table 3 shows the mean IPSS change from baseline at 24 months according to various categories of baseline characteristics. Symptom improvement in the WW-no treatment subgroup was similar across the different categories, with no obvious signals of a baseline characteristic that indicated better symptom outcomes. IPSS improvements generally appeared greater in the FDC group and WW-no treatment subgroup than in the WW-TAM subgroup and were similar in the FDC group and WW-no treatment subgroups, except in patients with the greatest degree of bother at baseline. Between baseline and month 24, the IPSS for patients with baseline BII of 0–3, 4–6 and 7–13 improved by 4.3, 5.4 and 7.1 points for patients in the FDC group, 5.0, 5.3 and 5.1 points for patients in the WW-no treatment subgroup and 1.6, 2.7 and 4.1 points in the WW-TAM subgroup (Fig. 2b).

## Discussion

Symptomatic improvements among men in the WW-no treatment subgroup appeared similar to those in men who received FDC therapy and better than those in the WW-TAM subgroup. There are a number of possible explanations for this. As previously reported [8], men who initiated tamsulosin tended to be older and have more bothersome symptoms at baseline. It is possible that younger men are better able to tolerate their symptoms; as a result, the degree of bother does not translate into changes in IPSS and treatment with tamsulosin is not indicated. By contrast, the threshold between the degree of bother and impact on IPSS may be lower in elderly men; i.e. although they may not have required medical intervention, the impact of symptoms on their general well-being may have led them to actively seek treatment. Another possible explanation relates to the study design. The WW-no treatment subgroup is composed of men who, from one study visit to the next, exhibited a lower score compared with baseline. CONDUCT selected a group of men in this arm who, perhaps by random choice, had a baseline symptom score higher than their true symptom status before enrolment. Over the course of the study, they never reached the high score recorded at baseline. Since all men whose symptom score stayed the same or increased were eliminated from this subgroup; this may have resulted in a remarkable (albeit artificial) ‘improvement’ that cannot be attributed to a placebo effect.

In the present post hoc analysis, symptom improvement was analysed according to age and BII at baseline. The data show that in terms of active treatment, FDC therapy appeared more effective than tamsulosin monotherapy in improving symptoms and lessening the degree of associated bother across all baseline subgroups. FDC therapy also offered an advantage over no treatment in younger patients, which seemed more apparent in men who were largely bothered by their symptoms at baseline (BII 7–13) or were less willing to spend the rest of their lives with the symptoms they had at baseline (IPSS-Q8 ≥ 4).

Patients with a higher BII at baseline also have a higher IPSS since the two measures are correlated. Among men who received immediate intervention with FDC, the absolute change in IPSS increased with increasing BII at baseline. This effect, however, was not observed among men in the WW-no treatment subgroup. The larger relative improvement in IPSS for men who received FDC treatment and had a BII score of 7–13 at baseline is a novel and unique insight provided by this post hoc analysis. Although further data are needed, these findings suggest that rather than symptom severity at baseline, the degree of associated bother (as determined by BII) may be a more relevant indicator as to whether a patient will benefit from treatment or not.

After initial symptoms develop, many patients will postpone a visit to the physician and try to adjust their lifestyle to self-manage their symptoms, only seeking medical advice, when their LUTS eventually become too bothersome [9]. Ensuring men are aware of the importance of lifestyle advice in managing their symptoms is therefore an important focus, and communication plays an essential role. For example, doctors who are able to spend more time with their patients may be more successful in motivating them to adopt lifestyle changes. The extent to which the advice was incorporated or adhered to in the CONDUCT is unknown. Further analyses to determine whether the social, educational or professional status of a patient affected the adoption of lifestyle changes would be useful.

There are several limitations that should be considered when interpreting these results. Study-related limitations include the open-label design and the lack of a placebo arm. Limitations of these analyses include their post hoc nature and the absence of formal statistical testing. As the analyses described herein were not powered to detect differences, data were provided for descriptive purposes only and must be interpreted accordingly. Of note, statistical analyses of subgroups often result in the misinterpretation or misuse of clinical data. Since the original randomisation no longer applies, newly defined subgroups are likely to be biased; furthermore, when multiple subgroup analyses are performed, the probability of a false positive finding can be substantial [10].

 In conclusion, some patients with moderately symptomatic BPH at risk of progression benefit from lifestyle advice alone. In this analysis, they are characterised by having a lower BII score at baseline; a novel finding that provides useful information for the care of patients and for future research. The CombAT study in men with moderate-to-severe symptoms showed symptomatic improvement with combination therapy, irrespective of BII [11]. Here, men with moderate symptoms but a higher bother score (7–13) appeared to have a substantial improvement in symptoms with combination therapy, even with lifestyle changes. The results of CONDUCT support guidelines that recommend conservative management for men with mild symptoms and those who are not bothered by moderate symptoms [4, 5] and suggest that using BII to further refine the selection of patients with moderate symptoms may provide a pathway to preventing the suboptimal management of BPH.

## Abbreviations

BII: BPH Impact Index
BPE: Benign prostatic enlargement
BPH: Benign prostatic hyperplasia
FDC: Fixed-dose combination
IPSS: International Prostate Symptom Score
LUTS: Lower urinary tract symptoms
PSA: Prostate-specific antigen
QoL: Quality of life
WW: Watchful waiting
